# 1,25-Dihydroxy vitamin D3 treatment attenuates osteopenia, and improves bone muscle quality in Goto-Kakizaki type 2 diabetes model rats

**DOI:** 10.1007/s12020-019-01857-5

**Published:** 2019-03-02

**Authors:** Yanlong Liang, Yanzhi Liu, Wenxiu Lai, Minqun Du, Shuhui Li, Limin Zhou, Yulin Mo, Pan Wang, Yalin Min, Liao Cui

**Affiliations:** 1 Guangdong Key laboratory for Research and Development of Natural Drugs, Department of Pharmacology, Guangdong Medical University, 524023 Zhanjiang, China; 2 Translational Medicine R&D Center, Institute of Biomedical and Health Engineering, Shenzhen Institutes of Advanced Technology, Chinese Academy of Sciences, 518055 Shenzhen, China

**Keywords:** Type 2 diabetes, Vitamin D3, Bone, Osteopenia, Calcitriol, GK rat

## Abstract

**Purpose:**

Osteopenia and skeletal fragility are considered to be the complications associated with type 2 diabetes mellitus (T2DM). The relationship between glucose metabolism, skeletal quality, and vitamin D have not been completely understood. We aimed to demonstrate a comprehensive bone quality profile in a T2DM model subject and to investigate whether 1, 25-dihydroxy vitamin D3 could prevent osteopenia and skeletal fragility in the diabetes model rats.

**Methods:**

Daily calcitriol (a 1, 25-dihydroxy vitamin D3 formulation, 0.045 μg/kg/day) treatment was administered to 21-week-old male *Goto-Kakizaki* (GK) rats (a genetic non-obese and non-insulin-dependent spontaneous diabetes rat model) for 20 weeks and the results were compared with those in untreated GK rats, and wild-type animals.

**Results:**

Micro-computed tomography, histomorphometry, and bone mineral density analysis demonstrated that T2DM induced significant osteopenia, and impairment of bone microarchitecture and biomechanical properties in GK rats. T2DM also significantly decreased bone formation and increased bone resorption parameters in three regions of the skeleton (proximal tibia, mid-shaft of the tibia, and lumbar vertebrae), and increased carboxy-terminal type I collagen crosslinks, tartrate-resistant acid phosphatase, muscle ubiquitin C, and bone thioredoxin interacting protein (TXNIP) expression. Calcitriol treatment significantly alleviated bone loss, and improved bone microarchitecture and biomechanical properties and also decreased serum glucose and glycated serum protein levels. Biomarkers of bone formation were significantly increased, while muscle ubiquitin C and bone TXNIP expression were significantly decreased following calcitriol treatment.

**Conclusions:**

These results suggest that 1,25-dihydroxy vitamin D3 treatment effectively attenuates osteopenia, and improves bone and muscle quality in GK type 2 diabetes model rats.

## Introduction

Type 2 diabetes mellitus (T2DM) is becoming an increasingly prevalent disease worldwide, according to the 2016 report of the World Health Organization [1], 422 million people worldwide have diabetes, and T2DM accounts for 90% of the cases [2]. Because of impaired signaling secondary to the binding of insulin to its receptor in T2DM, the activation of insulin-independent signaling proteins is attenuated in many tissues, including the liver, skeletal muscle, kidney, and bone [1–4], which leads to several serious consequences, particularly, osteoporosis [5–7]. Osteoporosis and fragility fractures have become increasingly recognized as skeletal complications of diabetes mellitus, especially, when these two complications occur in the middle-aged and elderly individuals with diabetes. In addition, T2DM not only impairs bone quality, but also induces a greater decline in the muscle mass, and muscle strength in elderly patients with type 2 diabetes when compared to that in age-matched normoglycemic controls [3, 4]. Age-related loss of muscle mass and strength is accelerated in type 2 diabetes patients. The increased type II muscle fiber atrophy in the elderly diabetic individuals could predispose to a great loss of muscle strength. Impaired bone quality and decreased muscle strength couple to induce a high incidence of fragility fractures in type 2 diabetic patients, which causes an enormous medical and economic burden. Therefore, the management and prevention of T2DM-induced bone fragility is a challenging issue. Despite evidence for increased bone fragility in type 2 diabetes patients, a comprehensive bone quality profile in a type 2 diabetes model subject has still not been fully presented. The protective strategy of osteoporosis treatment against the bone fragility in patients with type 2 diabetes also needs further investigation.

Vitamin D3 is well-known for its role in the maintenance of a healthy, mineralized skeleton through the regulation of calcium and phosphate homeostasis. Moreover, vitamin D may contribute to improved bone health independent of its role in calcium homeostasis. Several studies have reported that vitamin D is associated with several common diseases, including type 2 diabetes [5]. People with prediabetes and established diabetes have lower blood vitamin D_3_ concentrations than that in patients with normal glucose tolerance. Furthermore, in longitudinal observational studies, higher levels of vitamin D_3_ were found to be associated with lower rates of incident diabetes [6, 7].

The Goto-Kakizaki (GK) rat is a model of type 2 diabetes mellitus, produced originally by selective inbreeding for a hyperglycemic trait. These rats are characterized as having insulin resistance and an insulin secretory defect [8]. In the present study, we aimed to study a comprehensive bone quality profile in a type 2 diabetes model-GK rat and also to investigate whether 1, 25-dihydroxy vitamin D3 could prevent osteopenia and skeletal fragility and improve muscle quality in type 2 diabetes model rats.

## Methods

### Animals

Sixteen 10-week-old male GK rats (289.34 g ± 19.76 g) and eight age-matched Wistar rats (258.00 g ± 9.95 g) were obtained from Shanghai Slac Laboratory Animal Co. Ltd (Shanghai, China) and rats were bred to age 21-week-old before drug intervention experiment.

### Experimental protocols

GK rats at the age of 21-weeks were randomly allocated into two groups with eight rats per group and the eight age-matched male Wistar rats were used as the healthy control. The groups were: (1) healthy control (Control, Wistar rats); (2) Type 2 diabetes model (GK, Goto-Kakizaki Type 2 diabetes rats); (3) Treatment group (GK + Cal, GK rats were treated with calcitriol (R.P. Scherer GmbH &Co.KG, Germany, 0.045 μg/kg/day). Calcitriol administration continued for 20 weeks. The rats were sacrificed at age 41-weeks (21 + 20).

Rats were weighted and performed oral glucose tolerance test (OGTT) every 2 weeks. Rats were fasting 12 h over night before OGTT. According to the data from Shanghai Slac Laboratory Animal Co. Ltd on GK rats, rats with serum glucose levels above 7.0 mmol/L before glucose intake or above 11.1 mmol/L after 2 h by OGTT more than twice were considered to be diabetic. GK rats were all diagnosed with diabetes by the OGTT confirmation at the age of 13-weeks (data not shown). In vivo micro-computed tomography (μ-CT) analysis (Viva CT 40, Scanco, Switzerland) confirmed the GK rats began to show osteopenia compared to age-matched Wistar rats at the age of 18-weeks (data not shown).

All rats received subcutaneous injections with calcein (10 mg/kg, Sigma Chemical Co, USA, a bone formation surface marker) on days 3,4,13, and 14 before sacrifice on 20 weeks post initiated the calcitriol administration. The rats were sacrificed by cardiac puncture under anesthesia at the experimental endpoint. Serum was collected for biochemical marker assays. The pancreas and gastrocnemius were collected for histology analysis. The left proximal tibial metaphysis (PTM), cross-sections of the tibial shaft (TX), and the fourth lumbar vertebra (LV 4) were embedded in methylmethacrylate to obtain undecalcified sections, which were used for bone histomorphometry analysis. μ-CT analysis was used to analyze the trabecular changes on the proximal tibia. The right femurs were collected for the biomechanical properties analysis. The protein expression of TXNIP was determined with immunohistochemical analysis on the distal right femur. The expression of the ubiquitin C in gastrocnemius was detected by real-time PCR.

### Serum biomarker analyzes

Serum was collected and stored at −80 °C until analyzed. Serum alkaline phosphatase (AKP), carboxy-terminal collagen crosslinks (CTX)-1, calcium(Ca), phosphate (P), tartrate-resistant acid phosphatase (TRAP), osteocalcin (OCN), superoxide dismutase(SOD), malondialdehyde (MDA), glycated serum protein (GSP), and insulin (INS) were measured using commercially available assay kits (Nanjing Jiancheng Bioengineering Institute, Jiangsu, China) by following the manufacturer’s instruction.

### μ-CT analysis

μ-CT (Viva CT 40, Scanco Medical, Zurich, Switzerland) analysis was performed on left proximal tibia of the rats at the endpoint. Briefly, the scanning setting were in high resolution: X-ray energy 70 kVp, 114 μA, 8 W, integration time 200 ms. The region of interest (ROI) was in proximal tibial metaphysis (PTM) located between 1 and 3 mm distal to the growth plate–epiphyseal junction. Cortical bone was excluded from the measurement. Three-dimensional (3D) images were generated using a Gaussian filter (sigma 0.8, support 1). The 3D analysis was performed to determine bone volume/ tissue volume (BV/TV), connectivity density (Conn.D), trabecular number (Tb.N), trabecular separation (Tb.Sp), trabecular thickness (Tb.Th), and bone mineral density (vBMD).

### Bone histomorphometry analysis

The right proximal tibia metaphysis and the fourth lumbar vertebra were processed for undecalcified section and bone histomorphometric analyses. Frontal sections of 5 and 9 μm were obtained from each sample. The 5 μm sections were stained by Goldner’s trichrome and toluidine blue for static histomorphometric analyses. The 9 μm unstained sections were used for dynamic histomorphometric analyses. A semiautomatic digitizing image analysis system (Osteometrics, Inc., Decatur, GA, USA) was used for quantitative bone histomorphometric measurements. Briefly, the region of interest was located between 1 and 4 mm distal to the growth plate–epiphyseal junction. The quantitative analysis was performed on one section of each sample. The abbreviations of the bone histomorphometric parameters used were recommended by the American Society for Bone and Mineral Research Histomorphometric Nomenclature Committee [9]. All histomorphometric parameters and procedures were in accordance with previously published studies [10, 11].

### Biomechanical properties evaluation

The collected right femurs were isolated at the endpoint were then tested for mechanical properties (three-point bending test) using LLOYD LR5K Plus material testing system (Lloyd Instruments, Meerbusch, Germany). Each femur was placed on two lower supports that were 20 mm apart. Force was applied at the mid-diaphysis on the anterior surface so that the anterior surface was in compression and the posterior surface in tension. Load-displacement diagram for each test was recorded to determine structural strength properties (max load, fracture load, yield load and stiffness) for each specimen.

### Immunohistochemical and histological analysis

The immunohistochemical analysis of TXNIP expression on the decalcified femur was performed with an immunohistochemical kit (ZSGB-Biotechnology, Beijing, People’s Republic of China) according to manufacturer’s instruction and quantified by Image-Pro Plus 6.0 software (Media Cybernetics, Silver Spring, MD, USA) according to TXNIP staining. The anti-TXNIP antibody was obtained from Abcam (Cambridge, MA, USA). Gastrocnemius muscle was collected and weighted at the endpoint. Gastrocnemius and pancreas were embedded in paraffin and sectioned for histology analysis.

### Quantitative reverse transcription PCR (RT-PCR) analysis

Total RNA was extracted from the gastrocnemius muscle samples using TRIzol reagent (Invitrogen Life Technologies, Shanghai, China). Subsequently, complementary DNA was generated using a reverse transcriptase kit (Takara Bio, Inc., Otsu, Japan) according to the manufacturers’ instructions. The relative expression levels of ubiquitin C (Ubc) mRNA were determined using an SYBR Green real-time PCR kit (Takara Bio, Inc.) and normalized to GAPDH. RT-PCR analysis was performed using ABI 7500 Fast Real-Time PCR system (Applied Biosystems Life Technologies, Foster City, CA, USA) and the following gene-specific primers: Forward, 5′-AGGCAAGACCATCACTCTGG-3′ and reverse, 5′- CAAACCCAAGAACAAGCACA-3′ for Ubc. The primers were designed and synthesized by Shanghai Sangon Biotechnology Co. Ltd. (Shanghai, China).

### Statistical analysis

Data were presented as mean ± standard deviation. The statistical differences among groups were evaluated using SPSS 16.0 software (SPSS Inc., Chicago, IL, USA) by analysis of variance with Fisher’s protected least significant difference. A value of *P* < 0.05 was considered as statistically significant.

## Results

### GK diabetic rats developed marked bone loss and deterioration in bone quality

**Fig. 1:**
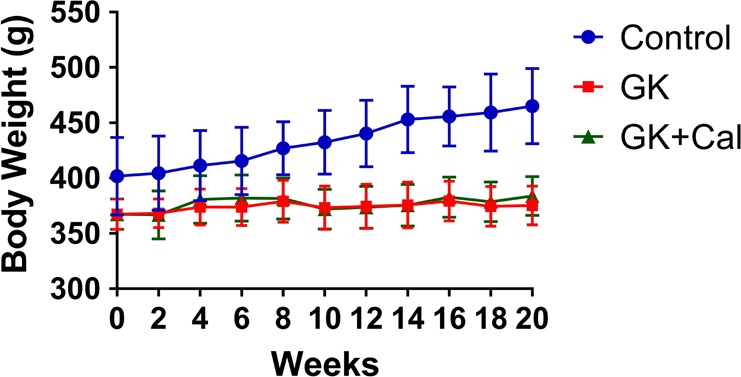
Body weight of GK diabetic rats with calcitriol treatment. Control, wild-type Wistar rats; GK, Goto-Kakizaki diabetic rats; GK + Cal, Goto-Kakizaki diabetic rats with calcitriol treatments

**Fig. 2:**
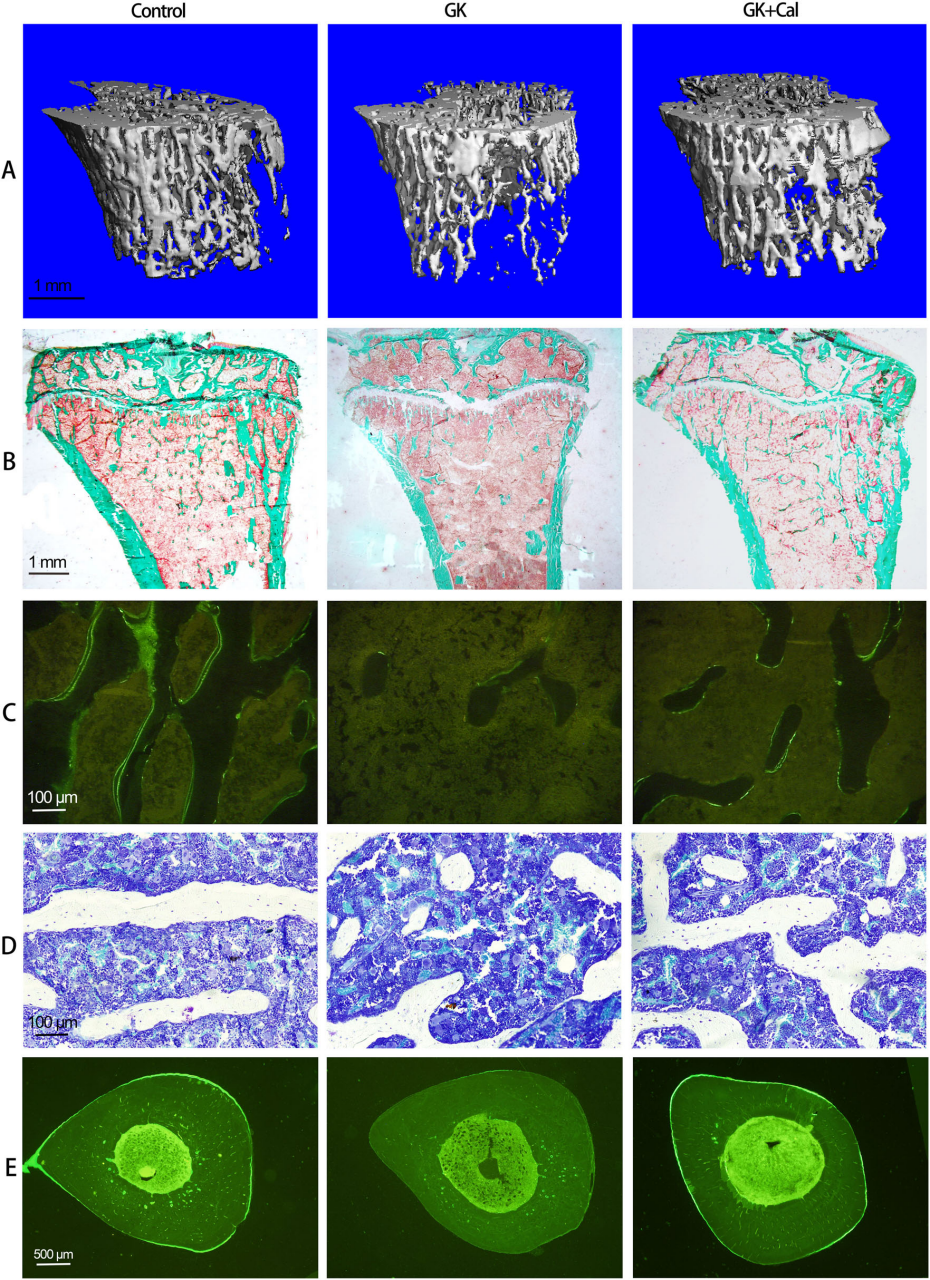
Representative PTM images of μ-CT and histomorphometry analysis in GK diabetic rats calcitriol treatment. **a** Representative 3D μ-CT images of the proximal tibial metaphysis (PTM); **b** Representative bone histomorphometry micrographs of PTM (undecalcified sections with Goldner’s trichrome staining); **c** Representative bone histomorphometry fluorescent micrographs of PTM (undecalcified sections with calcein labeling); **d** Representative bone histomorphometry micrographs of PTM (undecalcified sections with toluidine blue staining); **e** Representative bone histomorphometry fluorescent micrographs of tibial shaft (TX) (undecalcified sections with calcein labeling). Quantitative data were showed in Table 2 and Table 3. Control, wild-type Wistar rats; GK, Goto-Kakizaki diabetic rats; GK + Cal, Goto-Kakizaki diabetic rats with calcitriol treatments; PTM, proximal tibial metaphysis; CT, computed tomography

**Fig. 3:**
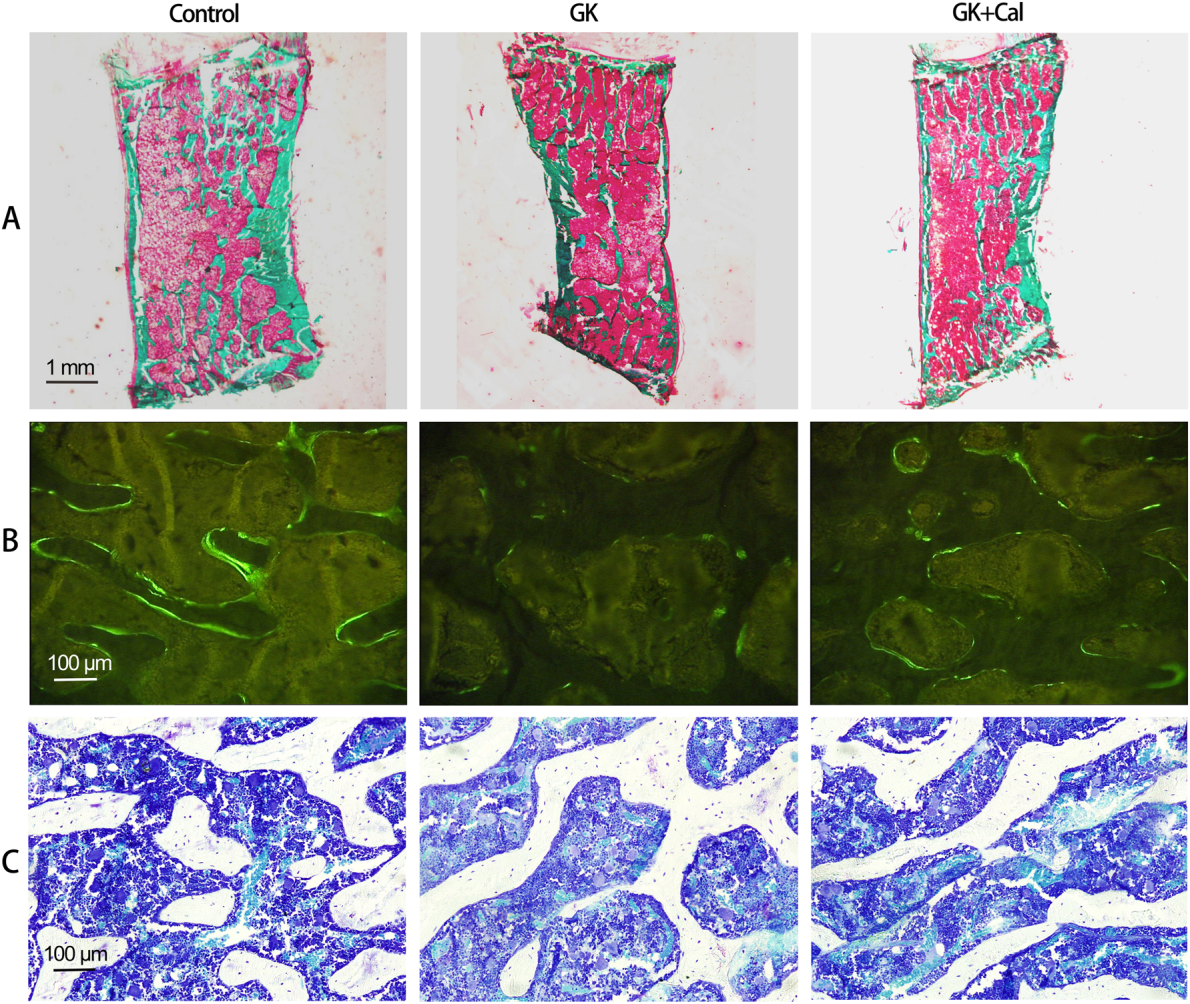
Representative lumbar vertebrae images of histomorphometry analysis in GK diabetic rats with calcitriol treatment. **a** Representative bone histomorphometry micrographs of lumbar vertebrae (undecalcified sections with Goldner’s trichrome staining); **b** Representative bone histomorphometry fluorescent micrographs of lumbar vertebrae (undecalcified sections with calcein labeling); **c** Representative bone histomorphometry micrographs of lumbar vertebrae (undecalcified sections with toluidine blue staining). Bone histomorphometry data of lumbar vertebrae was shown in Table 4. Control, wild-type Wistar rats; GK, Goto-Kakizaki diabetic rats; GK + Cal, Goto-Kakizaki diabetic rats with calcitriol treatments

**Fig. 4:**
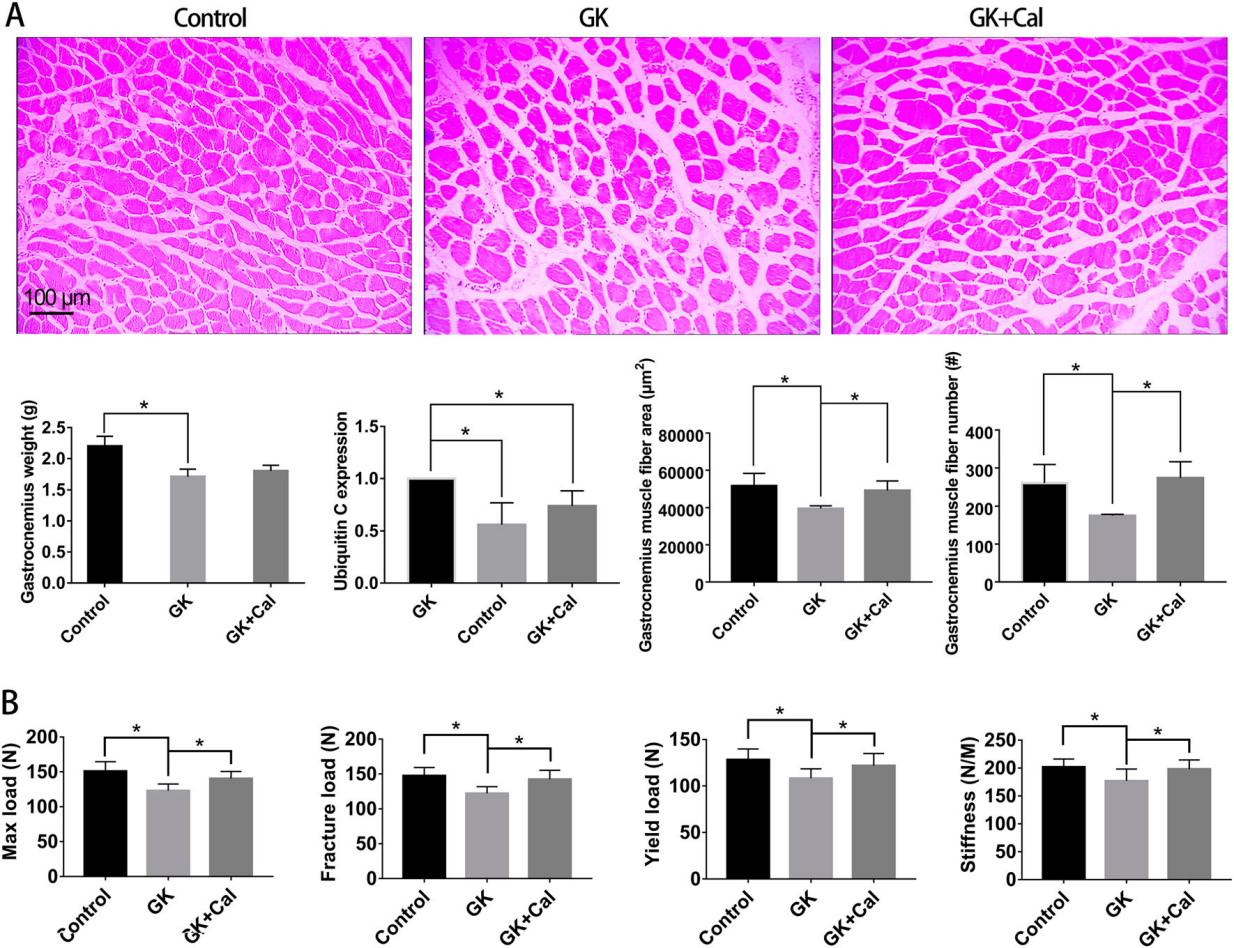
Gastrocnemius muscle analyses and bone biomechanical properties analyses of GK diabetic rats with calcitriol treatment. **a** Representative gastrocnemius histological images (H&E stain) and gastrocnemius muscle analysis in GK diabetic rats with and without calcitriol treatment; **b** Bone biomechanical properties analyses of GK diabetic rats with osteopenia undergoing calcitriol treatment. Notes: Vs. Control ^*^P < 0.05, vs. GK ^#^*P* < 0.05, Values are presented as mean ± SD. Control, wild-type Wistar rats; GK, Goto-Kakizaki diabetic rats; GK + Cal, Goto-Kakizaki diabetic rats with calcitriol treatments

**Fig. 5:**
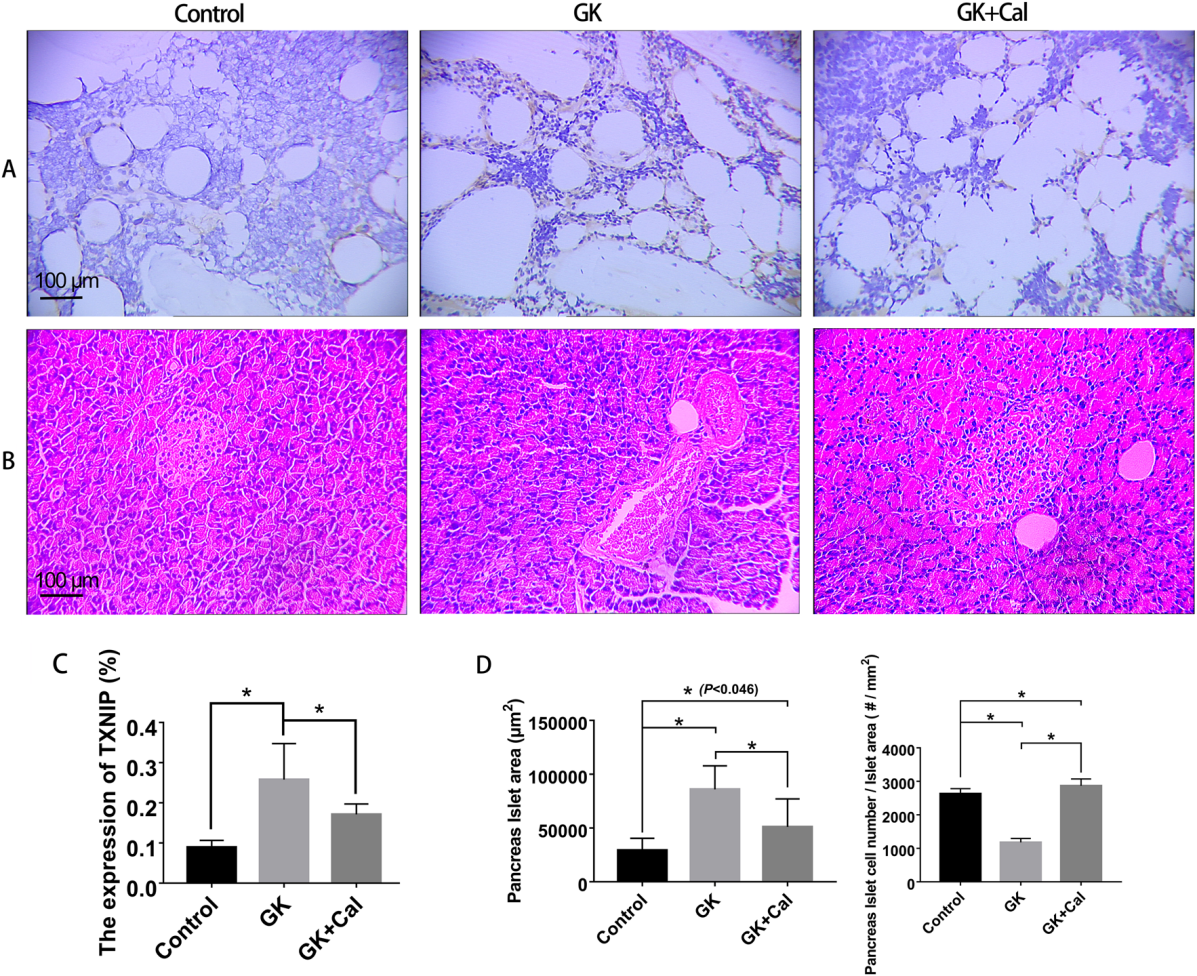
Immunohistochemical analysis of TXNIP expression of femur and pancreas pathology analysis in GK diabetic rats with calcitriol treatments. **a** Immunohistochemical analysis of TXNIP expression in the distal femurs. **b** Representative pancreas histological images (H&E stain); **c** Semi-quantitative results of TXNIP expression in the distal femurs. **d** Semi-quantitative data of pancreas islet in different groups. Notes: Vs. Control *P < 0.05, vs. GK ^#^*P* < 0.05, Values are presented as mean ± SD. Control, wild-type Wistar rats; GK, Goto-Kakizaki diabetic rats; GK + Cal, Goto-Kakizaki diabetic rats with calcitriol treatments

GK diabetic rats revealed significant lower body weight and higher serum glucose levels than those of wild-type controls (Fig. 1, Table 1, and Table 5). Serum biomarker and biochemical assays indicated that osteocalcin levels were significantly decreased whereas TRAP, CTX-I, and AKP levels were significantly increased in GK diabetic rats compared with controls. GK diabetic rats also demonstrated significantly higher levels of INS, GSP, MDA, with a lower *P* and SOD level in the serum. When compared with control, the data from μ-CT analysis indicated that the trabecular BV/TV ratio of GK rats decreased by 33% and was accompanied by significant deterioration in both bone geometry and microstructure parameters (Conn.D, trabecular number, trabecular thickness, trabecular separation, and vBMD) (Fig. 2 and Table 2). In the histomorphometry analysis, bone formation parameters including mineralizing surface, mineral apposition rate, and bone formation rate all significantly decreased in GK diabetic rats (Table 3). The results were found not only in trabecular bone but also in cortical bone. Bone histomorphometry analysis in the lumbar vertebra also showed consistent data as PTM (Fig. 3 and Table 4). Further, bone histomorphometric analysis revealed that the osteoblast surface ratio was significantly decreased whereas the osteoclast surface showed a mild increased compared to that in the controls. The deteriorated geometry, microstructure and density of bone directly led to a decrease in the bone quality of the GK rats. In terms of bone mechanical properties, apparent material strength (max load decreased by 18.5%, yield load decreased by 15.6%, and fracture load decreased by 17.1%) and structural strength (stiffness decreased by 12.3%) were significantly lower in GK diabetic rats than that in controls (Fig. 4b). Histological analyses suggested that the GK diabetic rats also demonstrated significant deteriorating pathological changes in the gastrocnemius muscle and pancreas. GK diabetic rats showed lower muscle fiber number and area, and enlarge pancreas islet with less islet cell density compared with controls. Ubiquitin C gene expression of gastrocnemius muscle and TXNIP protein expression were both significantly increased in GK diabetic rats (Figs. 4 and 5).

**Table 1:** Oral glucose tolerance test (OGTT) 2 h test per 2 weeks monitoring serum glucose change on GK diabetic rats post initiate calcitriol treatment

Drug intervetion	OGTT 0 h (mmol/L)			OGTT 1 h (mmol/L)			OGTT 2 h (mmol/L)		
Time (weeks)	Control	GK	GK + Cal	Control	GK	GK + Cal	Control	GK	GK + Cal
0	3.89 ± 0.15	7.08 ± 0.47*	6.03 ± 0.30*	5.61 ± 0.19	16.65 ± 1.40*	14.86 ± 1.34*	4.09 ± 0.11	11.53 ± 0.88*	11.19 ± 0.75*
2	3.90 ± 0.11	6.04 ± 0.30*	6.84 ± 0.32*	5.41 ± 0.24	16.22 ± 1.22*	15.50 ± 1.35*	4.10 ± 0.14	12.30 ± 1.26*	10.49 ± 0.86*
4	3.93 ± 0.12	6.19 ± 0.56*	6.11 ± 0.88*	5.48 ± 0.24	16.15 ± 0.73*	16.07 ± 1.59*	4.09 ± 0.12	12.89 ± 1.72*	10.91 ± 0.83*^,#^
6	3.95 ± 0.12	6.64 ± 1.08*	6.59 ± 0.61*	5.46 ± 0.34	16.34 ± 1.48*	15.03 ± 1.60*	4.13 ± 0.10	12.55 ± 1.46*	10.93 ± 0.85*^,#^
8	3.88 ± 0.13	6.38 ± 0.75*	7.00 ± 1.06*	5.30 ± 0.29	16.30 ± 1.27*	17.83 ± 2.43*	4.13 ± 0.10	11.88 ± 1.65*	10.93 ± 0.72*
10	3.90 ± 0.12	6.05 ± 0.48*	5.94 ± 0.41*	5.48 ± 0.21	16.38 ± 1.84*	13.54 ± 2.36*	4.18 ± 0.09	11.95 ± 2.04*	10.03 ± 1.49*
12	4.11 ± 0.43	5.86 ± 0.87*	6.11 ± 0.48*	5.36 ± 0.27	17.20 ± 1.32*	16.80 ± 2.79*	4.41 ± 0.40	12.81 ± 1.11*	10.96 ± 0.60*^,#^
14	4.28 ± 0.29	6.31 ± 0.53*	5.63 ± 0.49*^,#^	5.45 ± 0.35	16.95 ± 1.94*	15.31 ± 1.68*	4.60 ± 0.27	12.09 ± 0.86*	10.80 ± 0.88*
16	4.26 ± 0.18	6.63 ± 0.63*	5.72 ± 0.43*	5.39 ± 0.33	16.90 ± 1.38*	14.60 ± 0.96*^,#^	4.68 ± 0.21	12.34 ± 0.93*	10.74 ± 0.43*^,#^
18	4.21 ± 0.20	6.11 ± 1.03*	6.16 ± 0.52*	5.49 ± 0.24	18.26 ± 1.65*	13.86 ± 1.98*^,#^	4.61 ± 0.16	12.00 ± 1.07*	10.87 ± 2.91*
20	4.29 ± 0.22	6.44 ± 0.47*	5.41 ± 0.79*	5.54 ± 0.29	16.31 ± 1.84*	15.17 ± 2.11*	4.67 ± 0.19	11.95 ± 0.57*	9.36 ± 0.61*^,#^

Note: Vs. Control ^*^P < 0.05, vs. GK ^#^*P* < 0.05. Values are presented as mean ± SD

*Control* wild-type Wistar rats, *GK* Goto-Kakizaki diabetic rats, *GK+Cal* Goto-Kakizaki diabetic rats with calcitriol treatments

**Table 2:** μ-CT analysis of proximal tibial metaphysis (PTM) in GK diabetic rats with calcitriol treatment

Group	BV/TV (%)	Conn-D (1/mm^3^)	Tb.N (1/mm)	Tb.Th (mm)	Tb.Sp (mm)	vBMD (mg/cm^3^)
Control	18.00 ± 1.90	28.76 ± 4.89	2.47 ± 0.25	0.11 ± 0.008	0.41 ± 0.04	232.09 ± 13.95
GK	12.00 ± 3.10*	15.41 ± 4.18^*^	1.91 ± 0.36^*^	0.089 ± 0.004^*^	0.55 ± 0.11^*^	182.65 ± 21.05^*^
GK + Cal	17.00 ± 4.70^#^	23.97 ± 7.50^#^	2.46 ± 0.46^#^	0.10 ± 0.019^#^	0.43 ± 0.09	224.58 ± 30.32^#^

Note: Vs. Control *P < 0.05, vs. GK ^#^*P* < 0.05, Values are presented as mean ± SD

*Control* wild-type Wistar rats, *GK* Goto-Kakizaki diabetic rats, *GK+Cal* Goto-Kakizaki diabetic rats with calcitriol treatments, *μ-CT* micro-computed tomography, *PTM* proximal tibial metaphysis, *TV* tissue volume, *BV* bone volume, *Conn-D* connectivity density, *Tb.N* trabecular number, *Tb.Sp* trabecular separation, *Tb.Th* trabecular thickness, *vBMD* volumetric bone mineral density

**Table 3:** Bone histomophormetric parameter analysis of proximal tibial metaphysis (PTM) and tibial shaft (TX) on GK diabetic rats with calcitriol treatment

Group	Ob.S/B	Oc.S/BS	MS/BS	MAR	BFR/BS	BFR/BV	BFR/TV	Ct.Ar	Ct.Ar	Ma.Ar	E-L.Pm	P-L.Pm	P-MAR	P-BFR/BS
	(%)	(%)	(%)	(μm/day)	(%/year)	(%/year)	(%/year)	(mm^2^)	(%)	(%)	(%)	(%)	(μm/day)	(%/year)
Control	0.99 ± 0.26	0.58 ± 0.10	12.35 ± 3.83	1.17 ± 0.15	14.42 ± 4.74	147.77 ± 45.30	16.78 ± 4.50	5.13 ± 0.15	82.04 ± 0.98	17.96 ± 0.96	21.82 ± 6.04	51.70 ± 8.80	13.47 ± 2.00	47.4 ± 12.44
GK	0.49 ± 0.06*	0.63 ± 0.07	5.53 ± 1.32*	0.60 ± 0.08*	3.34 ± 1.02*	45.78 ± 13.19*	2.72 ± 0.96*	3.98 ± 0.15*	67.16 ± 1.67*	32.84 ± 0.96*	11.57 ± 1.09*	15.73 ± 4.54*	5.95 ± 1.71*	5.15 ± 1.32*
GK + Cal	0.70 ± 0.10*^,#^	0.58 ± 0.09	7.83 ± 0.89*^,#^	0.80 ± 0.64^#^	6.27 ± 1.06*^,#^	70.32 ± 8.56*^,#^	7.06 ± 0.66*^,#^	4.12 ± 0.31*	68.28 ± 1.96*	31.72 ± 1.96*	17.07 ± 0.77*^,#^	31.17 ± 3.91*^,#^	9.26 ± 1.15*^,#^	17.22 ± 4.85*^,#^

Note: Vs. Control *P < 0.05, vs. GK ^#^*P* < 0.05. Values are presented as mean ± SD

*Control* wild-type Wistar rats, *GK* Goto-Kakizaki diabetic rats, *GK+Cal* Goto-Kakizaki diabetic rats with calcitriol treatments, *PTM* proximal tibial metaphysis, Bone histomophormetric parameters of PTM include: *Ob.S* osteoblast surface, *Oc.S* osteoclast surface, *BS* bone surface. *MS/BS* mineralizing surface, *MAR* mineral apposition rate, *BFR* bone formation rate, *BS* bone surface, *BV* bone volume, *TV* tissue volume, *B.Ar/T.Ar* trabecular bone area ratio in tissue area, *Tb.N* trabecular number, *Tb.Sp* trabecular separation, *Tb.Th* trabecular thickness. *TX* tibial shaft, Bone histomophormetric parameter of TX include: cortical bone area, Ct.Ar; cortical bone area ratio in tissue area, Ct.Ar(%); bone marrow area ratio in tissue area, Ma.Ar(%); *E-L.Pm* endocortical single-labeled surface ratio in bone surface, *P-L.Pm* periosteal single-labeled surface ratio in bone surface, *P-MAR* periosteal mineral apposition rate, *P-BFR* periosteal bone formation rate, *BS* bone surface

**Table 4:** Bone histomorphometric parameter analysis of lumbar vertebrae on GK diabetic rats with calcitriol treatment

Group	B.Ar/T.Ar (%)	Tb.Th (μm)	Tb.N (1/mm)	Tb.Sp (μm)	Ob.S/BS (%)	Oc.S/BS (%)	MS/BS (%)	MAR (μm/day)	BFR/BS (%/year)	BFR/BV (%/year)	BFR/TV (%/year)
Control	0.25 ± 0.03	81.03 ± 9.90	3.13 ± 0.22	240.10 ± 19.76	1.08 ± 0.06	0.61 ± 0.04	5.15 ± 0.93	0.79 ± 0.12	6.73 ± 1.74	51.26 ± 15.51	12.76 ± 3.34
GK	0.19 ± 0.02*	70.21 ± 7.94*	2.67 ± 0.10*	304.71 ± 13.55*	0.56 ± 0.06*	0.67 ± 0.05*	2.03 ± 0.49*	0.34 ± 0.023*	1.27 ± 0.22*	11.02 ± 1.48*	2.06 ± 0.36*
GK + Cal	0.22 ± 0.02*^,#^	74.81 ± 1.60^#^	2.94 ± 0.15^#^	265.95 ± 18.73*^,#^	0.79 ± 0.02*^,#^	0.64 ± 0.03	4.80 ± 0.74^#^	0.56 ± 0.09*^,#^	2.65 ± 0.34*^,#^	21.57 ± 2.68*^,#^	4.76 ± 0.78*^,#^

Note: Vs. Control *P < 0.05, vs. GK ^#^*P* < 0.05. Values are presented as mean ± SD

*Control* wild-type Wistar rats, *GK* Goto-Kakizaki diabetic rats, *GK+Cal* Goto-Kakizaki diabetic rats with calcitriol treatments, *B.Ar/T.Ar* trabecular bone area ratio in tissue area, *Tb.N* trabecular number, *Tb.Sp* trabecular separation, *Tb.Th* trabecular thickness, *Ob.S* osteoblast surface, *Oc.S* osteoclast surface, *BS* bone surface, *MS/BS* mineralizing surface, *MAR* mineral apposition rate, *BFR* bone formation rate, *BV* bone volume, *TV* tissue volume

### Calcitriol treatment restored bone formation, suppressed bone resorption, and increased bone and muscle quality in GK diabetic rats

As observed on μ-CT analysis, calcitriol treatment significantly increased the mass (trabecular BV/TV, trabecular number), vBMD, and bone microarchitecture parameters when compared to those in untreated GK diabetic rats (Fig. 2 and Table 2). As to the bone histomorphometric analysis, calcitriol treatment significantly increased bone formation parameters; including mineralizing surface, mineral apposition rate, and bone formation rate in both trabecular and corticoid bone (Table 3). Additionally, calcitriol treatment also significantly increased osteoblast surface and reduced osteoclast surface. Bone biomechanical data revealed a significant higher max load (increased by 14.1%), yield load (increased by 12.7%), fracture load (increased by 16.4%) and stiffness (increased 11.9%) in GK diabetic rats with calcitriol treatment than in untreated GK diabetic rats (Fig. 4). Serum biomarkers demonstrated that osteocalcin was significantly increased whereas TRAP and CTX-I were significantly decreased in calcitriol-treated GK diabetic rats compared with untreated GK diabetic rats (Table 5). Calcitriol treatment significantly decreased TXNIP protein expression and mildly decreased MDA in GK diabetic rats compared with untreated GK diabetic rats. Additionally, gastrocnemius weight loss, the decreased muscle fiber area and number, as well as the decreased ubiquitin C gene expression in the gastrocnemius muscle were significantly alleviated by calcitriol treatment (Fig. 4). High levels of serum glucose INS and GSP in GK diabetic rats were also decreased by calcitriol treatment (Table 5). Calcitriol treatment suppress the deterioration of pancreas islet and increased islet cell density in GK rats.

**Table 5:** Serum biochemical marker analyses of GK diabetic rats with calcitriol treatment

Group	INS (IU/L)	GSP (mmol/L)	OCN (ng/mL)	AKP (king unit/100 mL)	CTX-I (ng/mL)	TRAP (IU/L)	SOD (U/L)	MDA (nmol/mL)	Ca (mmol/L)	*P* (mmol/L)
Control	22.76 ± 3.43	0.28 ± 0.04	6.60 ± 0.89	12.94 ± 1.84	1.56 ± 0.25	16.98 ± 2.40	323.80 ± 10.68	14.64 ± 1.34	1.89 ± 0.53	2.14 ± 0.28
GK	31.57 ± 2.85*	0.46 ± 0.06*	3.97 ± 0.73*	55.20 ± 14.04*	2.74 ± 0.39*	29.67 ± 5.22*	293.90 ± 9.49*	16.54 ± 1.38*	1.99 ± 0.46	1.85 ± 0.31*
GK + Cal	33.47 ± 1.81*	0.30 ± 0.05^#^	4.56 ± 0.99*	29.48 ± 10.24*^,#^	2.02 ± 0.14*^,#^	23.08 ± 5.61*	293.50 ± 9.16*	15.71 ± 2.31	2.20 ± 0.16*	2.38 ± 0.36^#^

Note: Vs. Control ^*^P < 0.05, vs. GK ^#^*P* < 0.05. Values are presented as mean ± SD

*Control* wild-type Wistar rats, *GK* Goto-Kakizaki diabetic rats, *GK+Cal* Goto-Kakizaki diabetic rats with calcitriol treatments. *INS* insulin, *GSP* glycated serum protein, *OCN* osteocalcin, *AKP* serum alkaline phosphatase, *CTX-I* carboxy-terminal collagen crosslinks, *TRA* tartrate-resistant acid phosphatase

## Discussion

In the present study, we have demonstrated that GK rats showed higher serum glucose, INS and GSP levels and deteriorating pathological changes in the pancreas, which may mimic cardinal symptom of T2DM. Our data demonstrated that T2DM significantly impaired bone formation and increased bone resorption, and, further, induced deterioration in the bone microarchitecture and biomechanical properties. We also showed that bone deterioration occurs at multiple skeletal sites (tibia and lumbar vertebrae), where both trabecular and cortical bone are present. Additionally, T2DM caused increased muscle loss and degradation that further impaired physical functioning of the musculoskeletal system in GK rats. Previous studies have demonstrated skeletal changes in GK rats by dual X-ray absorptiometry (DXA), peripheral quantitative CT (PQCT) [12], and histomorphometric and biomechanical analysis [13]. Our results were not only consistent with those of previous studies but also add to those findings with comprehensive data, including bone histomorphometry; μ-CT; biomechanical properties; pathology; and immunohistochemical, muscle, and serum biomarker analyses in GK rats.

On the other hand, our data also demonstrated that 20 weeks calcitriol (1,25-dihydroxy vitamin D3) intervention increased bone formation and bone mass in the trabeculae of the tibia and lumbar vertebrae, as well as in cortical bone, which was consistent with the results of the μ-CT. Calcitriol also mildly suppressed bone resorption and osteoclast activity. The increased bone mass and improved microarchitecture of bone in calcitriol-treated GK rats eventually yielded better biomechanical properties than those in untreated GK rats. Muscle loss and degradation in GK rats were alleviated by calcitriol treatment. Calcitriol treatment brings better bone and muscle quality that together contribute to reducing fracture risk in diabetic rats. Previous researches have confirmed that vitamin D3 plays an important role within the bone remodeling process and it has been used in clinical practice for the prevention of disorders associated with bone health [14–16]. In addition, calcitriol decreased serum glucose, and GSP and attenuated pancreas damage in GK T2DM model rats when compared to its effect in untreated GK rats, which suggest calcitriol not only has a beneficial role in improving bone and muscle health but also is involved in glucose metabolism.

An increasing number of clinical studies have investigated the involvement of vitamin D3 supplementary treatment in reducing the risk fractures induced by diabetes. There is evidence that vitamin D insufficiency is an independent risk factor for both type 1 diabetes (T1D) and T2DM in adults [17, 18]. It has been observed that people with prediabetes and established diabetes have lower blood vitamin D concentrations than do patients with normal glucose tolerance levels. Epidemiological data have demonstrated that the active vitamin D3 levels in patients with T2DM is significantly lower than that in normal healthy people; therefore, vitamin D3 and calcium supplementation will help to protect the function of cells in the pancreatic islet of patients with T2DM and decrease related complications in T2DM [19]. Previous researchers have investigated whether vitamin D supplementation has a causal effect on glucose homeostasis and incident diabetes, ultimately leading to an improvement in the glucose homeostasis and preventing diabetes [20–22]. Our data revealed that calcitriol treatment could effectively improve the impaired glucose tolerance, and reduce the serum glucose level, and attenuate the pathological damage of pancreatic islet cells in the T2DM GK rats. Our results are consistent with those of previous studies that vitamin D3 can improve insulin resistance and lower serum glucose [23–25]. However, although calcitriol treatment significantly decreased the glucose level in GK rats when compared to that of GK untreated rats, the glucose levels in calcitriol-treated rats were still >twofolds higher than that in healthy control rats. Some clinical trials have reported that vitamin D supplementation has no significant effects or only mild effects on the prevention and treatment of diabetes [26]. These results suggest that calcitriol treatment could not substantially prevent the progression of diabetes.

Previous studies revealed that vitamin D3 improved muscle strength and strengthened the body balance capability in elderly people, thereby decreasing the risk of falling [27]. Skeletal muscle is an important target organ of T2DM. Diabetes is often accompanied by symptoms of severe muscle atrophy. Based on pathological conditions, muscle atrophy is mainly attributed to muscle protein metabolism disturbances [28]. Catabolism of protein in the skeletal muscle cell mainly follows three degradation pathways: the lysosome pathway, the mitochondrial protease pathway, and the ubiquitin protease pathway [29]. Our results demonstrated that calcitriol treatment significantly increased gastrocnemius weight and suppressed ubiquitin C expression in GK rats, and this could lead to the suppression of muscle atrophy and improvement in muscle quality.

Oxidative stress is one of the pivotal risk factors for osteoporosis, and is involved in almost all kinds of osteoporosis (for example, osteoporosis induced by estrogen deficiency, aging, glucocorticoids, and diabetes) [30]. Oxidative stress induced by reactive oxygen species (ROS), which increases with aging or with the onset of a pathological state, can adversely affect bone homeostasis; this is the immediate cause of suppression of the generation and survival of osteoclasts, osteoblasts, and osteocytes [31]. Oxidative stress is increased in T2DM and this appears to underlie the development of T2DM diabetic complications [32]. Our serum data indicated that calcitriol treatment decreased MDA in GK rats accompanied by decreased TXNIP expression. Researchers have found that TXNIP protein is a key protein that could amplify oxidative stress in the initial stage of the diabetic individuals. The upregulated protein will stimulate oxidative stress and lead to the death of pancreatic insulin-producing cells. TXNIP also affects osteocalcin secretion by osteoblasts, and induces bone resorption by regulating OPG/RANKL pathway [33]. In this study, we demonstrated that TXNIP expression was significantly increased in GK rats, and calcitriol treatment could suppress TXNIP expression in GK diabetic rats. The results suggested that calcitriol treatment may improve bone quality in GK rats by suppressing TXNIP-mediated oxidative stress.

In conclusion, our study demonstrated that calcitriol treatment (1, 25-dihydroxy vitamin D3) effectively attenuates osteopenia, improves bone and muscle quality in GK type 2 diabetes model rats.
